# Ketamine alters oscillatory coupling in the hippocampus

**DOI:** 10.1038/srep02348

**Published:** 2013-08-02

**Authors:** Fábio V. Caixeta, Alianda M. Cornélio, Robson Scheffer-Teixeira, Sidarta Ribeiro, Adriano B. L. Tort

**Affiliations:** 1 Brain Institute, Federal University of Rio Grande do Norte, Natal, RN 59056-450, Brazil; 2 Edmond and Lily Safra International Institute of Neuroscience of Natal, Natal, RN 59066-060, Brazil

## Abstract

Recent studies show that higher order oscillatory interactions such as cross-frequency coupling are important for brain functions that are impaired in schizophrenia, including perception, attention and memory. Here we investigated the dynamics of oscillatory coupling in the hippocampus of awake rats upon NMDA receptor blockade by ketamine, a pharmacological model of schizophrenia. Ketamine (25, 50 and 75 mg/kg i.p.) increased gamma and high-frequency oscillations (HFO) in all depths of the CA1-dentate axis, while theta power changes depended on anatomical location and were independent of a transient increase of delta oscillations. Phase coherence of gamma and HFO increased across hippocampal layers. Phase-amplitude coupling between theta and fast oscillations was markedly altered in a dose-dependent manner: ketamine increased hippocampal theta-HFO coupling at all doses, while theta-gamma coupling increased at the lowest dose and was disrupted at the highest dose. Our results demonstrate that ketamine alters network interactions that underlie cognitively relevant theta-gamma coupling.

Oscillations in the activity of neuronal populations are associated with the coordination of distributed neuronal groups believed to underlie cognitive processing^1^,^2^,^3^. Disturbance in cortical oscillations has been suggested as a possible neural basis for symptoms of mental disorders such as schizophrenia^4^,^5^. In particular, aberrant gamma-frequency oscillations (30–100 Hz) have been reported in schizophrenic patients^6^,^7^, but alterations in other frequency bands are also likely to play a role^8^.

Acute blockade of glutamate N-methyl-D-aspartate receptors (NMDAR) by ketamine in humans induces negative and positive symptoms similar to those found in schizophrenia^9^, and exacerbates its core symptoms when administered to patients^10^. In animals, acute NMDAR blockade induces behavioural, biochemical and electrophysiological alterations^11^,^12^,^13^,^14^,^15^ that present predictive, constructive and face validity as a pharmacological model for schizophrenia^16^. Previous studies in rodents have shown that ketamine increases the power of gamma^12^,^15^ and delta^17^ oscillations, and may differentially affect theta power depending on recording region^15^,^18^,^19^. Of note, some of the electrophysiological alterations induced by ketamine, such as aberrant gamma oscillations, have been dissociated from its motor effects^14^.

Brain rhythms of different frequencies are not independent, but can rather interact in several ways^20^. Cross-frequency coupling (CFC) among neuronal oscillations has been linked to brain functions such as detection of sensory signals, reward signalling, decision-making, working memory, attention and learning (see ref. 21 for a review). CFC patterns differ across brain areas^22^,^23^ and change dynamically in a task-relevant manner in response to sensory, motor and cognitive events^21^,^24^. Although CFC has been implied in several brain functions, few studies have attempted to characterize CFC in schizophrenia^25^ or in its animal models^13^.

In this work we investigated the effects of acute NMDAR blockade by ketamine on the dynamics of spectral content and oscillatory interactions in the hippocampus, a brain region that has long been associated with the schizophrenia phenotype^26^. We focused on cognitively relevant frequency bands: theta (5–10 Hz), gamma (30–100 Hz) and high-frequency oscillations (HFO; 110–160 Hz). We found that ketamine leads to frequency- and region-specific alterations of local field potential (LFP) power, altered phase synchrony, and aberrant cross-frequency coupling of neural oscillations. Taken together, these results demonstrate that ketamine distorts normal oscillatory interactions in the rat hippocampus.

## Results

### NMDAR blockade increases locomotion and high frequency oscillations

**Figure 1:**
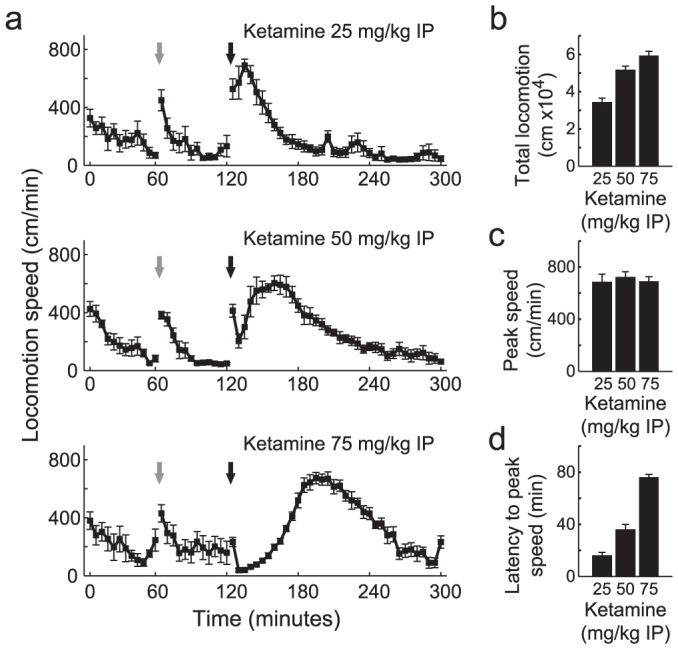
Acute injection of sub-anaesthetic doses of ketamine induces hyperlocomotion in rodents*.* (a) Freely moving rats received intraperitoneal (IP) injections of saline and ketamine (25 mg/kg – n = 6; 50 mg/kg – n = 7; 75 mg/kg – n = 5) at 60 and 120 min after the beginning of the recording session, respectively, and were monitored for additional 180 min. In this and all other figures, grey and black arrows denote saline and ketamine injections, respectively. Locomotion speed was monitored at 30 frames per second and averaged over 5-min blocks among all animals from each group. Notice that ketamine induces an increase in locomotion speed at all doses. (b–d) Total locomotion (b), peak speed (c), and latency to peak locomotion speed (d) after ketamine injection are shown for each dose. Data are shown as mean ± SEM over animals.

**Figure 2:**
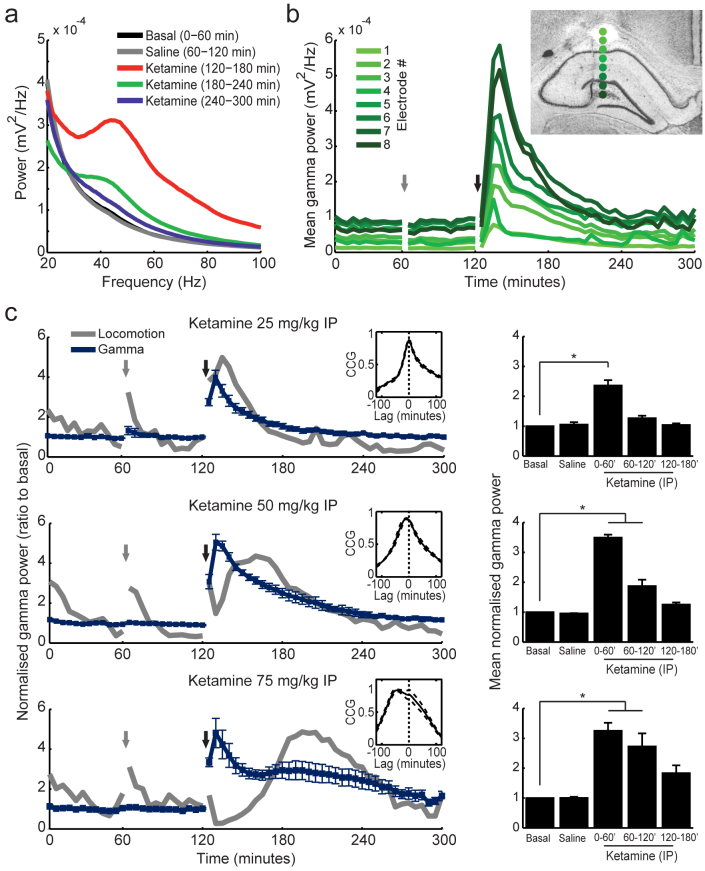
Ketamine-induced increase in gamma power can be temporally dissociated from its effect on locomotor activity. (a) Representative power spectra in an animal treated with 50 mg/kg ketamine IP (mean over all 8 electrodes; 5-Hz moving average smoothing). (b) Time-course of mean gamma power (30–100 Hz) for all electrodes in the same animal as in (a). Inset shows histology with estimated electrode depths (indicated by green dots at the right of the lesion). Grey and black arrows denote saline and ketamine injections, respectively. (c) Left: Group results of normalised gamma power variations (blue) induced by three doses of ketamine (different rows, as labelled). Grey line depicts mean locomotion speed in arbitrary units (see Fig. 1a for actual units). Insets show cross-correlograms between normalised gamma power and locomotor activity. Right: Mean normalised gamma power in 1-hour blocks, as labelled. *p<0.001 (repeated measures ANOVA) followed by Bonferroni post-hoc test. Data are shown as mean ± SEM over animals.

Consistent with previous reports^11^,^12^,^14^, we found that systemic administration of ketamine increased locomotor activity (Fig. 1a, b) and gamma power (Fig. 2) at all doses studied. While peak locomotion speed was similar for all doses (Fig. 1c), higher ketamine doses were associated with greater latency to peak locomotor activity (16, 36 and 76 minutes, respectively; Fig. 1d), mainly due to transitory ataxia.

In Fig. 2a we show the gamma band power averaged across all electrodes in a representative animal treated with ketamine. Peak gamma power occurred during the first hour post-ketamine injection, and approached baseline levels three hours afterwards. In Fig. 2b we show the time-course of gamma power variations in each of the 8 electrodes in the same animal (Fig. 2b inset); notice that gamma power increases along the CA1-dentate gyrus axis, as previously reported^27^. The increase in gamma power induced by ketamine was apparent in all recording sites; in fact, baseline normalised gamma power provided similar time-courses for all electrodes in the bundle (data not shown). At the group level, in contrast to the time-course of locomotor activity (Fig. 1a), gamma power peaked within 5–10 minutes after ketamine injection at all doses studied (Fig. 2c). These results show that the time-course of locomotor and electrophysiological alterations caused by NMDAR blockade may be dissociated (see cross-correlations in Fig. 2c insets and ref. 14).

**Figure 3:**
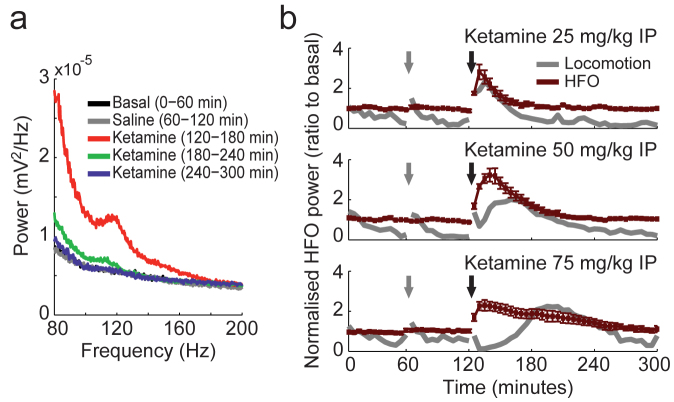
Hippocampal high-frequency oscillations (HFO: 110–160 Hz) increase during acute NMDAR blockade by ketamine. (a) Representative power spectra of a *stratum oriens* electrode in an animal treated with 50 mg/kg ketamine IP. (b) Group results of normalised HFO power variations (red) induced by three doses of ketamine (different rows, as labelled). Grey line depicts mean locomotion speed in arbitrary units. Grey and black arrows indicate saline and ketamine injections, respectively. Data are shown as mean ± SEM over animals.

We found that ketamine also increased hippocampal HFO power, with a similar time-course to the increase in gamma oscillations (see Fig. 3a for a representative electrode and Fig. 3b for group results). Incidentally, it has recently been shown that ketamine increases HFO activity in motor cortex, nucleus accumbens, and other basal ganglia nuclei^28^,^29^, suggesting that abnormally high levels of HFO may be a widespread effect of NMDAR blockade.

### NMDAR blockade modulates hippocampal theta oscillations in a layer-dependent manner

**Figure 4:**
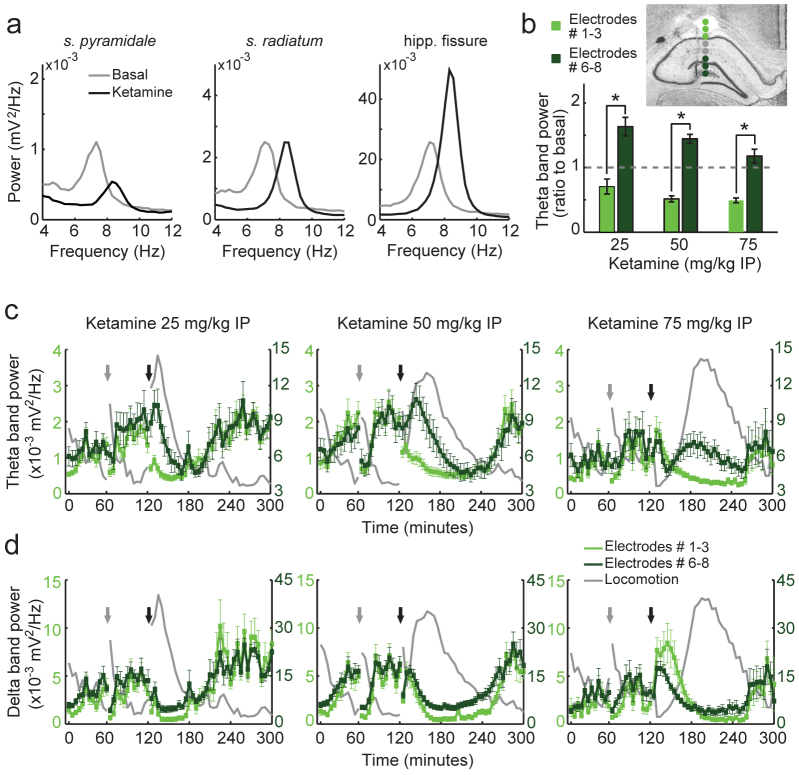
Ketamine differently affects theta oscillations in different layers of the hippocampus. (a) Power spectral densities during 25-min of baseline recordings (grey) and during 25-min after administration of 50 mg/kg ketamine IP (black) for three electrodes simultaneously recorded from a linear bundle in a representative animal. (b) Group results of mean theta band (5–10 Hz) power recorded simultaneously in two subsets of 3 electrodes (inset) during a 5-min epoch of peak locomotion induced by ketamine, normalised by the mean theta power during baseline (dashed line); *p<0.001 (*t*-test). (c, d) Time-course of mean theta (c) and delta (d) power in the two subsets of electrodes located in different hippocampal layers (see the inset in b for estimated electrode locations). Grey and black arrows indicate saline and ketamine injections, respectively. Mean locomotor activity is also shown in grey (arbitrary units). Notice that different y-axis scales are used for each subset of electrodes to facilitate comparison. Data are shown as mean ± SEM over electrodes.

Along with high-frequency alterations, we found that low-frequency LFP signals were also modulated by NMDAR blockade. In Fig. 4a we show the power spectral density in the theta range of three electrodes recorded simultaneously from an animal during pre- and post-injection of 50 mg/kg ketamine IP. Notice in this example that while theta band power decreased after ketamine injection in *stratum pyramidale*, it did not change in *stratum radiatum*, and was markedly increased at the hippocampal fissure. Notice further that theta peak frequency was shifted in all recording sites from 6–8 Hz during drug-free locomotion periods to 7–10 Hz following ketamine injection, probably due to increases in locomotion speed. Fig. 4b shows group results for theta band power modulation during peak locomotion induced by NMDAR blockade in a subset of electrodes located in *stratum* *oriens-alveus* and *pyramidale* (Electrodes #1–3) and in another subset located in *stratum lacunosum-moleculare*, hippocampal fissure and dentate gyrus (Electrodes #6–8). The mean power spectral density is displayed in Supplementary Fig. S1 online. Ketamine differentially affected theta band power in the two subsets of electrodes at all doses studied, and this effect occurred specifically during hyperlocomotion (Fig. 4c).

A recent study showed that ketamine IP at the dose of 50 mg/kg – but not 20 mg/kg – increases the power of hippocampal delta (1–4 Hz) oscillations during a period of ~15 minutes following the injection^17^ (see also ref. 19). Since a greater level of delta power can potentially lead to a greater area under the curve of the power spectral density in the theta range (spectral leakage^30^) even in the absence of a theta peak, we next investigated whether putative changes in delta power could account for the changes in theta power reported above. As shown in Fig. 4d, delta power was highly modulated by locomotion speed; in particular, delta power was high during periods of low locomotion preceding saline and ketamine injections, and also after the hyperlocomotion episode had ceased (Fig. 4d). Thus, the apparent high levels of theta band power in these periods (Fig. 4c) are actually due to spectral leakage from delta power and do not correspond to a genuine theta activity. Consistent with a previous report^17^, the doses of 50 and 75 mg/kg transiently increased delta power, which returned to basal levels before the peak of ketamine-induced hyperlocomotion activity (Fig. 4d), i.e., when the layer-dependent variations in theta power were most striking (Fig. 4c). Moreover, contrary to theta, delta power time-course was qualitatively similar in all electrodes (Fig. 4d). Therefore, ketamine-induced alterations in delta power cannot account for the dichotomy in theta modulation across the CA1-dentate gyrus axis observed during hyperlocomotion.

### NMDAR blockade leads to increased phase synchrony in multiple high-frequency bands

**Figure 5:**
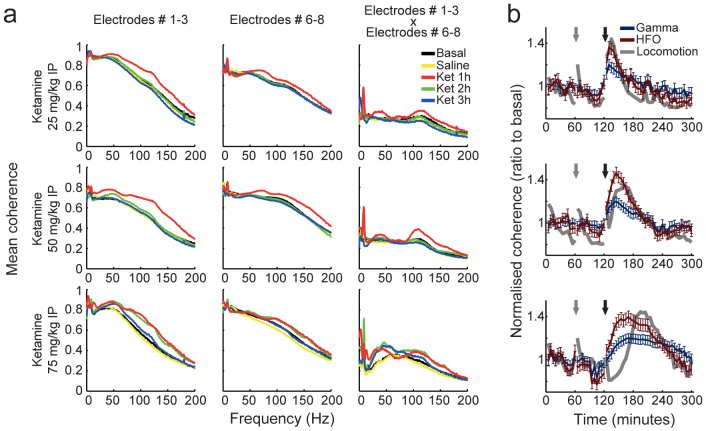
Ketamine induces hypersynchrony in multiple frequency bands in the hippocampus. (a) Phase coherence spectra before and after treatment with different doses of ketamine (rows) for different electrode pair combinations (columns). (b) Time-course of normalised phase coherence in the gamma and HFO bands (mean over all electrode pairs in all animals). Mean locomotor activity is also shown in grey (arbitrary units). Data are shown as mean ± SEM. Gamma and HFO coherence was significantly increased during the first hour post-ketamine at all doses, and also during the second hour at the highest dose (p<0.01; repeated measures ANOVA followed by Bonferroni post-hoc test). Ket = ketamine, HFO = high-frequency oscillations (110–160 Hz).

We next investigated the levels of phase coherence across recording sites, and found that ketamine induced transient hypersynchrony in a wide range of fast LFP oscillations from 30 to 200 Hz (Fig. 5a). Phase coherence spectra were typically multimodal, exhibiting peak values in the traditional gamma range, as well as in frequencies above 100 Hz. The changes in coherence induced by ketamine were observed among electrode pairs located in multiple hippocampal depths (Fig. 5a). Interestingly, electrode pairs located at *stratum lacunosum-moleculare* and dentate gyrus presented coherence peaks in a faster gamma frequency than electrode pairs located at *stratum* *oriens-alveus* and *pyramidale*. Peaks in HFO phase coherence were particularly prominent for electrodes pairs across hippocampal layers.

In Fig. 5b we show time-courses of mean phase coherence changes in the gamma and HFO bands across all electrode pairs. Gamma and HFO phase coherence increased immediately after ketamine injection and only returned to baseline values after the hyperlocomotion episode ended. Interestingly, the relative increase in phase coherence induced by ketamine was much more prominent for HFO than gamma oscillations. In all, these results show that ketamine alters inter-site synchrony of multiple frequency bands in the hippocampus.

### NMDAR blockade alters cross-frequency coupling

We next examined the effects of ketamine on the coupling between low- and high-frequency LFP oscillations. Typically, low frequency phase modulates the amplitude of higher frequency oscillations^22^. This type of oscillaory interaction is deemed to be involved in cognitive processing (see ref. 21 for a review). Consistent with previous reports^23^,^24^,^31^, we found prominent CFC in most CA1 electrodes; theta phase strongly modulated the amplitude of HFO in electrodes located above the pyramidal layer (i.e., *stratum oriens-alveus*), while the amplitude modulation of high-gamma (HG; 60–100 Hz) was maximal in electrodes located in *stratum lacunosum-moleculare* and hippocampal fissure^23^. Finally, in spite of the low-gamma (30–60 Hz) power increase depicted in Fig. 2a, we did not find prominent coupling between theta and low-gamma in CA1, as reported previously^23^.

**Figure 6:**
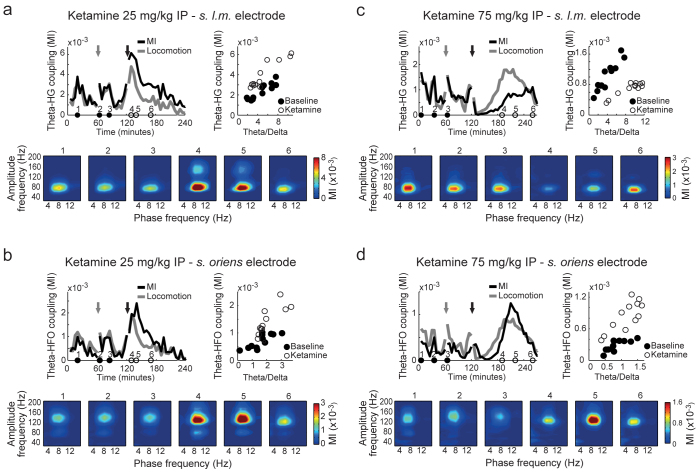
Ketamine alters cross-frequency coupling of neuronal oscillations. (a) Top left: time-course of theta-HG coupling strength and mean locomotion speed (arbitrary units) before and after treatment with 25 mg/kg ketamine IP. Top right: Scatter plot of theta-HG coupling as a function of theta/delta ratio for each 5-min time block (black: pre-ketamine; white: post-ketamine). Bottom: comodulation maps obtained from the 5-min epochs indicated in the top left panel by black (pre-) and white (post-ketamine) circles. The results were obtained from an electrode in *stratum lacunosum-moleculare* presenting prominent theta-HG coupling in a representative animal. (b) Same as in (a), but for an electrode in *stratum oriens* presenting prominent theta-HFO coupling in the same animal. (c, d) Same as (a) and (b), but for an animal treated with 75 mg/kg ketamine IP. HG = high-gamma (60–100 Hz), HFO = high-frequency oscillations (110–160 Hz), MI = modulation index.

**Figure 7:**
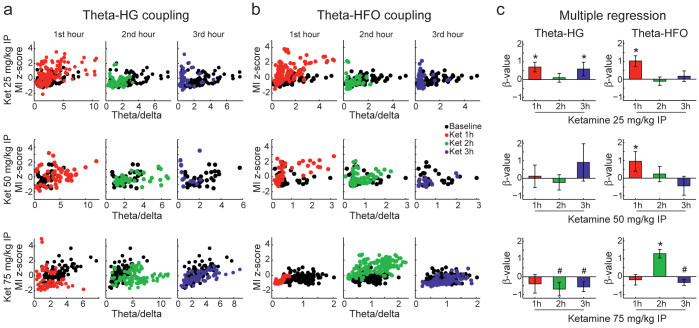
Ketamine alters cross-frequency coupling (group results). (a) Scatter plots of theta-HG normalised coupling strength in 5-min epochs for all analysed electrodes as a function of theta/delta power ratio (black = pre-ketamine, red = 1^st^ hour post-ketamine, green = 2^nd^ hour, blue = 3^rd^ hour). (b) Same as in (a), but for theta-HFO coupling. Lower x-axis limits in (b) compared to (a) are due to the fact theta-HFO and theta-HG coupling occur mostly above and below the pyramidal layer, respectively^23^, which have different theta/delta power ratios. (c) Multiple regression coefficients (β-values) for changes in coupling strength before and after ketamine controlling for the level of theta/delta power ratio. A non-zero β-value indicates that CFC level is significantly altered by ketamine independent of changes in the theta/delta ratio (*p<0.01). Only electrodes presenting theta-HG or theta-HFO coupling in baseline comodulation maps were used in these analyses (see Methods and Supplementary Fig. S4 online). Total number of electrodes analysed for theta-HFO coupling for each dose was 13 (25 mg/kg), 18 (50 mg/kg), and 10 (75 mg/kg), and for theta-HG coupling 15 (25 mg/kg), 16 (50 mg/kg) and 10 (75 mg/kg). Ket = ketamine, HG = high-gamma (60–100 Hz), HFO = high-frequency oscillations (110–160 Hz), MI = modulation index.

To illustrate the effect of acute NMDAR blockade on CFC, for each example in Fig. 6 we show six comodulation maps computed for 5-min time blocks before and after ketamine injection (as indicated by black and white dots in the top left panel, respectively). CFC strength for all time blocks is shown in the top right panel of each example. These results are representative for recording sites with theta-HG (Fig. 6a, c) and theta-HFO coupling (Fig. 6b, d) for the lowest (Fig. 6a, b) and highest (Fig. 6c, d) ketamine dose. Surprisingly, we found that ketamine had a differential effect on theta-HG coupling depending on dose: while the lowest dose increased theta-HG coupling (Fig. 6a), the highest dose disrupted this oscillatory interaction (Fig. 6c; see also Fig. 7a for group results). On the other hand, ketamine increased theta-HFO coupling at all doses (Fig. 6b, d and Fig. 7b).

Since CFC strength typically varies with theta power^23^,^24^,^31^, we next investigated whether the results above could be related to ketamine effect on theta oscillations. To that end, we plotted mean CFC strength as a function of the theta/delta power ratio (Fig. 7a, b). We note that due to the spectral leakage of delta power into the theta rage that occurs during periods of immobility (c.f. section above), the theta/delta ratio is a better measure of genuine theta activity in the LFP than the mean power in the theta range. Moreover, the theta/delta ratio is a spectral measure highly correlated with locomotion speed (Supplementary Fig. S2 online), and thus also serves to investigate whether changes in CFC strength are explained by changes in locomotion. We found that ketamine altered CFC in a similar way as described above even after controlling for this confounding factor (see Fig. 7c for multiple regression analyses). These results therefore show that acute NMDAR blockade alters CFC in a frequency-specific and dose-dependent way.

### NMDAR blockade does not alter the distribution of electrical dipoles in the hippocampus

**Figure 8:**
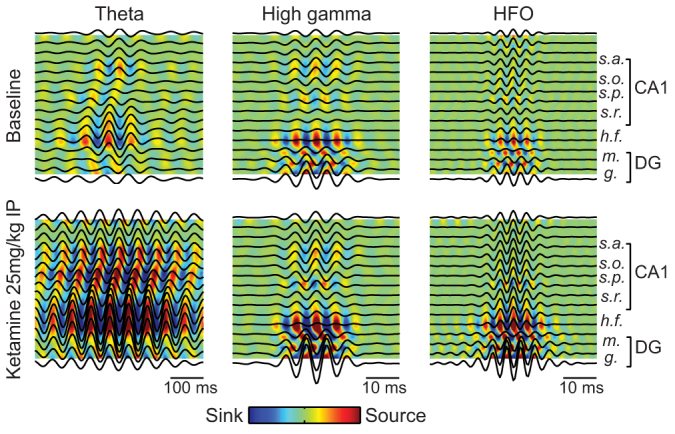
Ketamine does not alter dipole distribution in the hippocampus. Triggered LFP averages and current source density (CSD) analysis from a 16-site probe (100-μm spacing) recorded from an animal subjected to 25 mg/kg ketamine IP. Dark lines indicate LFP averages triggered by the peaks of filtered LFP signals. Colour plots show the associated CSD maps. For each frequency (theta: 5–10 Hz; HG: 60–100 Hz; HFO: 110–160 Hz), colour scaling is the same before and after ketamine. Electrode 2 was used as the reference electrode in all analyses. Notice that the position of sinks and sources remain unaltered after drug administration for all frequencies analysed. Estimated recording sites are indicated at the right.

Finally, we performed current source density (CSD) analysis in one additional animal. Baseline CSD plots (Fig. 8, top row) for the different frequency ranges were similar to those previously described^27^,^30^. We found that ketamine did not alter the spatial distribution of sinks and sources pairs (Fig. 8, bottom row). These results indicate that NMDAR blockade alters pre-existing hippocampal oscillations but does not generate new dipoles. Further, these analyses indicate that the oscillations investigated in this work are generated in the hippocampus, and not volume conducted from other brain regions.

## Discussion

In this study we showed that acute sub-anaesthetic doses of ketamine alter the cross-frequency interaction between theta phase and the amplitude of two higher frequency rhythms in the hippocampus: high-gamma and HFO. In addition, we also found that ketamine increases gamma and HFO power, alters oscillatory phase synchrony, and differentially modulates theta power in a layer-specific manner.

Consistent with previous studies^12^,^14^,^15^, we found altered behaviour and increased hippocampal gamma power during acute blockade of NMDAR. While low doses of ketamine cause correlated increases in locomotor activity and total gamma power, higher doses can induce different time-courses of behavioural and electrophysiological alterations. In fact, ketamine also increases gamma oscillations in sedated and anesthetised animals^14^. These observations indicate that alterations in gamma power and hyperlocomotion are two independent effects of NMDAR blockade. While hyperlocomotion induced by acute NMDAR blockade is currently considered a predictive model of positive schizophrenic symptoms^16^, the dissociation between gamma activity and hyperlocomotion suggests that altered gamma oscillations may have additional translational significance^32^.

Theta oscillations are believed to serve as a temporal organizer for a variety of functions, such as sensorimotor integration^33^ and coordination of cell assemblies by means of phase modulating gamma oscillations^2^. Consistent with recent findings^15^, here we found that acute NMDAR blockade differently alters theta power depending on hippocampal layer. These findings show that different theta dipoles have different sensitivities to NMDAR blockade. Entorhinal cortex inputs give rise to the theta dipole in *stratum lacunosum-moleculare*^34^. A greater theta activity in this layer following NMDAR blockade may thus be associated with an overflow of sensory information from the entorhinal cortex to the hippocampus.

Neuronal synchrony has been proposed to play a role in dynamically selecting and routing information within and across brain structures^1^. This hypothesis gave rise to the idea that abnormal synchrony would underlie symptoms of cognitive disorders such as autism and schizophrenia^4^. However, whether schizophrenia is associated with increased or decreased neuronal synchrony remains an open question^5^. Previous studies found reduced inter-trial phase coherence (ITC) in schizophrenic patients^6^,^35^, typically accompanying a decrease in stimulus-evoked gamma power^35^. It should be noted that ITC measures the level of phase resetting following a sensory stimulus within a recording site, and not the level of phase locking between LFP oscillations recorded from different sites, as studied here. Regarding the latter, positive schizophrenia symptoms may be associated with increased connectivity^7^,^36^. Recent evidence suggests that although schizophrenic patients have reduced evoked gamma power, they could have abnormally high levels of basal gamma power^36^,^37^. If confirmed, these findings would solve current inconsistencies between animal models (which show increased levels of gamma power and synchrony) and human studies (which point to reduced evoked gamma power and synchrony; for discussion, see ref. 37). Altogether, our and other results suggest that psychotic symptoms caused by NMDAR hypofunction are associated with an over-processing of information through functionally hyper-connected structures. Therefore, like in other brain disorders such as Parkinson disease and epilepsy^4^, pathological hypersynchrony could also play a role in schizophrenia.

Theta-gamma coupling has been hypothesised to form a neural coding system that allows the representation of multiple items in a sequential order^38^. Abnormalities in theta-gamma coupling have been thus suggested as a possible electrophysiological substrate of disordered thoughts and impaired working memory^8^,^38^. Also, it should be noted that recent CFC studies have demonstrated that theta modulates multiple higher frequency bands, which occur within (30–100 Hz) and beyond (>100 Hz) the traditional gamma band^22^,^23^,^24^,^39^. For instance, theta preferentially modulates high-gamma (60–100 Hz) in CA1 and low-gamma (30–60 Hz) in CA3^22^,^24^,^40^. Additionally, theta phase also modulates higher frequency activity circumscribed into the 110–160 Hz band in *stratum oriens-alveus*^23^,^39^, which we refer to as HFO. Interestingly, ketamine leads to a significant increase in HFO activity in the nucleus accumbens^28^, a limbic region implicated in schizophrenia that receives massive connections from the hippocampus^41^. This suggests that higher frequency oscillations above the gamma range may also be altered in schizophrenia.

Here we showed that ketamine increases theta-HFO coupling during peak locomotion at all doses studied, while its effect on theta-gamma coupling was dose dependent. Importantly, none of these effects can be explained by changes in theta power occurring during hyperlocomotion. The cognitive implications of increased theta-HFO coupling remain to be better understood, as well as the functional role of HFO per se^23^,^28^,^39^. A recent study has shown that physiological theta-HFO coupling significantly increases during REM sleep^31^. REM sleep is a brain state associated with incongruous thoughts and dreams. Many similarities have been pointed out between REM sleep and psychosis^42^, leading some to suggest that psychotic symptoms would be associated with intrusion of a dreaming state into an awake mind^43^,^44^. While these suggestions remain to be appropriately tested, the finding of enhanced theta-HFO coupling during REM sleep^31^ and following NMDAR blockade (present results) supports such a view.

Theta-gamma coupling, on the other hand, increased with the lowest dose of ketamine but was disrupted with the highest dose. Current theories on the combined function of theta and gamma oscillations suggest that disrupting their coupling would lead to deficits in brain functions such as working memory^38^. However, the functional implications of increased theta-gamma coupling upon lower levels of NMDAR blockade are harder to interpret. It may be that increased oscillatory power, synchrony and cross-frequency coupling are all correlates of an aberrant state of brain hyperexcitability and altered information flow, which could underlie dysfunctions such as hallucinations and flight of ideas.

Recent findings suggest that dysfunction of GABAergic interneurons are likely to underlie the electrophysiological alterations reported here^45^. Accordingly, ablation of NMDAR in parvalbumin (PV) positive interneurons in mice leads to enhancements of basal gamma activity^46^,^47^. Moreover, ketamine does not induce hyperlocomotion in these knockout mice^47^, suggesting a critical involvement of the blockade of NMDAR in PV interneurons for the manifestation of positive schizophrenic symptoms. NMDAR ablation in corticolimbic interneurons has also been associated with the negative symptoms of the disease^48^. These findings help link together the NMDA hypofunction hypothesis of schizophrenia with the alterations of GABAergic interneurons seen in post-mortem studies of schizophrenic subjects^49^. In addition to PV+ interneurons, hypofunction of NMDAR in oriens lacunosum-moleculare (OLM) interneurons could also be involved in the pathophysiology of schizophrenia^50^,^51^. These cells synapse on distal portions of the apical dendrites of pyramidal cells, where projections from the entorhinal cortex arrive^50^. A hypofunction of OLM cells would thus favour entorhinal cortex inputs^52^, which could then lead to increased theta oscillations in *stratum* *lacunosum-moleculare*, as observed here. GABAergic interneurons are also likely to underlie coupling between theta and gamma rhythms^50^,^53^, and would thus further mediate aberrant CFC patterns following NMDAR blockade. In all, a preferential action of NMDA antagonists on inhibitory cells is compatible with increased levels of excitation^54^,^55^ and altered neuronal oscillations.

Building a bridge between electrophysiological findings in animal models and schizophrenic patients has proven to be a challenge^5^. A large part of the alterations described here, particularly at the HFO band, would not have been noticed by scalp EEG because of its frequency band limitations. Data obtained by invasive techniques, such as electrocorticograms, are unfortunately scarce in schizophrenia. In addition, antipsychotic drugs by themselves cause oscillatory changes^32^, and are therefore important confounding factors in clinical studies. Thus, while at variance with some previous human studies^6^,^35^, our results add to others^7^,^36^,^37^ in the suggestion that some symptoms of the schizophrenia syndrome are mediated by an aberrant state of brain hyperactivity, including increases in the activity of fast oscillations, phase synchrony and cross-frequency coupling.

## Methods

### Surgical implantation of electrodes

Animal care and surgery procedures were approved by the Edmond and Lily Safra International Institute of Neuroscience of Natal Committee for Ethics in Animal Experimentation (permit 02/2011). Eight male Wistar rats (2–3 months old, 280–380 g) were used in the experiments. Seven animals were chronically implanted in the left dorsal hippocampus with one electrode bundle consisting of 8 vertically staggered tungsten microwires (50-μm diameter). Electrodes were aligned and spaced by 250 μm, spanning from CA1 *stratum oriens-alveus* to the *hilus* of the dentate gyrus (deepest electrode in AP: −3.6 mm, ML: −2.5 mm, DV: −3.5 mm). One additional animal was implanted with a 16-site probe across the left hippocampus (NeuroNexus Technologies; site area: 703 μm^2^; separation: 100 μm; impedance: 1–1.5 MΩ; location: AP: −3.6 mm, ML: −2.5 mm). All recordings were referenced to an epidural screw electrode implanted in the right parietal bone.

### Experimental procedures

After recovering for 7–10 days, animals were individually habituated to the recording room for 3 days. Experiments consisted of video and electrophysiological recordings of freely moving rats in a rectangular arena (50 × 4 × 40 cm) placed in a dimly lighted room. Recordings consisted of 3 stages: animals were first allowed to explore the arena for one hour (*basal*); then were injected with saline and recorded for another hour (*saline*); finally, animals received a single ketamine injection (Ketamina Agener®, 100 mg/ml, Agener União, Embu-Guaçu, SP) of either 25 (n = 6 rats), 50 (n = 7 rats) or 75 mg/kg (n = 5 rats) and were recorded for additional three hours (*ketamine*). All injections were intraperitoneal (IP). Depending on the stability of the recordings, each rat received up to 3 different doses separated by at least 3 days.

It should be noted that ketamine effects vary widely across species^56^: while 1–4 mg/kg of intravenous (IV) ketamine induces deep anaesthesia in humans, 20 mg/kg of IV ketamine induces only hypnosis in rats^57^. Used in isolation, the reported anaesthetic dose of ketamine in rats is 200 mg/kg IP^55^. Also, IP administration is far less effective than subcutaneous (SC) injections^15^.

### Electrophysiological recordings

Continuous recordings were performed using a multi-channel acquisition processor (MAP, Plexon Inc). Local field potentials (LFPs) were pre-amplified (1000×), filtered (0.7–300 Hz), and digitised at 1000 Hz. Electrode placement in CA1 was confirmed by inspecting coronal brain sections stained with cresyl violet, and by assessing responses evoked by perforant path stimulation (single pulse, 500 μA) along with other standard electrophysiological parameters such as presence of ripple oscillations and multi-unit activity at the pyramidal cell layer, theta phase reversal across *stratum radiatum*, and maximal theta power at the hippocampal fissure^27^.

### Behavioural analysis

Animals were video-recorded at 30 frames/second. Tracking of the animals position was made using MouseLabTracker (http://www.neuro.ufrn.br/incerebro/mouselabtracker.php), an open-source MATLAB version of a previously described software^58^. In order to avoid measuring small movements such as head and tail movements only displacements ≥2.0 mm/frame were considered. Locomotor activity was binned into 5-min blocks.

### Data analysis

Analyses of electrophysiological data were performed in MATLAB (MathWorks).

### Filter settings and extraction of the instantaneous phase and amplitude

Filtering was obtained using a linear finite impulse response filter by means of the *eegfilt* function from the EEGLAB toolbox (http://sccn.ucsd.edu/eeglab/), which applies the filter forward and then again backwards to ensure that phase delays are nullified. The instantaneous amplitude and phase time series of a filtered signal were computed from the analytical representation of the signal based on the Hilbert transform (*hilbert* function, Signal Processing Toolbox).

### Spectral analyses

Power spectra estimation was done by means of the Welch periodogram method using the *pwelch* function from the Signal Processing Toolbox (50% overlapping 4-s Hamming windows). The mean power over frequency ranges of interest was calculated for each electrode individually, then averaged across electrodes and animals. Phase coherence was calculated using the multitaper method by means of the *coherencysegc* function from the Chronux toolbox^59^ (http://chronux.org/) with parameters TW = 3 and K = 5 tapers, and window length of 4 seconds. Phase coherence was averaged from all electrode pairs in all animals. Power and phase coherence time-courses were obtained by averaging their values in 5-min blocks.

### Estimation of phase-amplitude coupling and comodulation maps

To assess phase-amplitude CFC, we used the Modulation Index (MI) recently described^22^,^24^. This index measures coupling strength between two frequency ranges of interest: a phase-modulating (*f_p_*) and an amplitude-modulated (*f_A_*) frequency. The comodulation map is obtained by expressing the MI for several frequency band pairs (4-Hz bin width with 2-Hz steps for *f_p_*, and 10-Hz bin width with 5-Hz steps for *f_A_*) in a bi-dimensional pseudocolour plot (see Supplementary Fig. S3 online for an illustrative example). Comodulation maps were computed using 5-min long LFPs recorded from single electrodes; only time windows associated with robust theta oscillations were used. Mean CFC strength between two frequency ranges was obtained by averaging the corresponding MI values; for example, mean theta-HG coupling corresponds to the average of MI values in the (4–10 Hz) × (60–100 Hz) region of the comodulation map, and similarly for theta-HFO coupling. We only computed theta-HG and theta-HFO coupling strength for electrodes that had theta-HG and theta-HFO coupling in the comodulation map, respectively (see Supplementary Fig. S4 online). Recording sites that did not present clear CFC in the comodulation map, or which the comodulation map revealed spike contamination^30^,^60^ (common in recordings from the CA1 pyramidal layer^30^ and dentate gyrus^23^), were not taken into account in further analyses (see Supplementary Fig. S4 online for representative examples of discarded electrodes). Consistent with recent reports^23^,^31^, theta-HFO coupling was mainly present in electrodes in *stratum oriens-alveus*, and theta-HG coupling from the CA1 pyramidal layer to *stratum lacunosum-moleculare*/hippocampal fissure.

### Triggered LFP averages and current source density (CSD)

Probe signals were amplified (200×), filtered (1 Hz–7.5 kHz), and digitised at 25 kHz (RHA2116, Intan Technologies). LFP averages were obtained by first filtering the LFP signal into the frequency ranges of interest; the amplitude peaks of each band were then identified and used for averaging 500-ms epochs centred at these timestamps. CSD analysis was obtained by -A+2B-C for adjacent sites. We used 60-s periods of prominent theta oscillations in these analyses.

### Statistics

Group means were compared by *t*-test for independent samples or by repeated measures ANOVA followed by Bonferroni post-hoc test. Multiple regression was performed to study changes in CFC level corrected for changes in locomotion and theta activity (as assessed by the theta/delta ratio; see Supplementary Fig. S2 online).

## Author Contributions

F.V.C., A.M.C. and R.S.-T. collected the data, A.B.L.T. and F.V.C. conceived the experiments and analysed the results, F.V.C., S.R. and A.B.L.T. wrote the paper.

## Supplementary Material

Supplementary Information Supplementary Figures and Legends
