# Supplementary material for: Ketamine alters oscillatory coupling in the hippocampus
Source: Sci Rep. 2013 Aug 2;3:2348. doi: 10.1038/srep02348 (PMC3731648; doi:10.1038/srep02348)
Supplement: Supplementary Information — Supplementary Figures and Legends [file srep02348-s1.pdf]

Ketamine alters oscillatory coupling in the hippocampus

Fábio V. Caixeta[1,2], Alianda M. Cornélio[1], Robson Scheffer-Teixeira[1], Sidarta Ribeiro[1], Adriano B.L. Tort[1]

1 - Brain Institute, Federal University of Rio Grande do Norte, Natal, RN 59056-450, Brazil
2 - Edmond and Lily Safra International Institute of Neuroscience of Natal, Natal, RN 59066-060, Brazil

SUPPLEMENTARY INFORMATION

4 Supplementary Figures + Legends

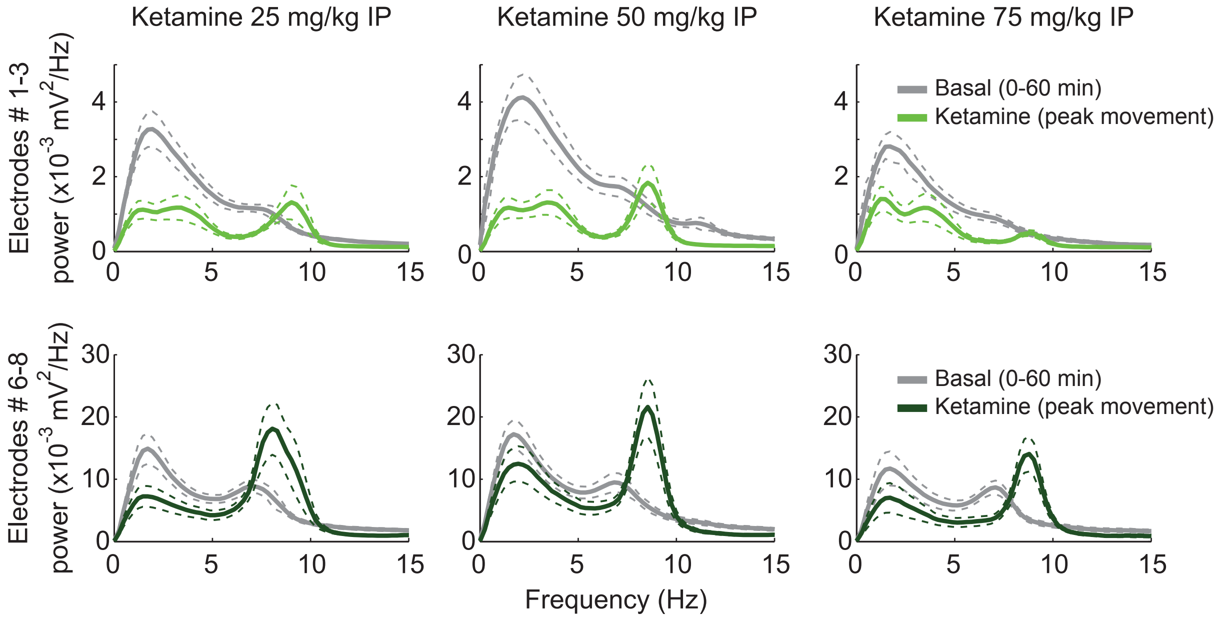

Supplementary Figure S1. Ketamine induced alterations of low frequency power depend on anatomical location. Group average power spectra during baseline and during peak hyperlocomotion induced by ketamine for electrodes located in *stratum oriens-alveus* and *pyramidale* (top row) and in *stratum lacunosum-moleculare*, hippocampal fissure and dentate gyrus (bottom row). Data are shown as mean ± SEM over electrodes.

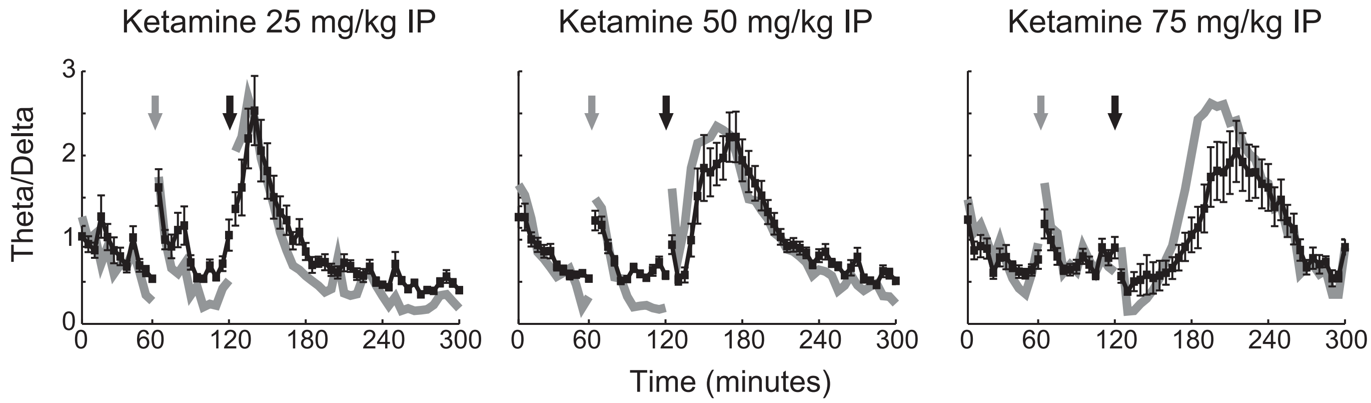

Supplementary Figure S2. Theta/delta power ratio is associated with locomotor activity. Dark lines indicate time-course of theta/delta ratio averaged for different ketamine doses. Grey line depicts mean locomotion speed in arbitrary units (see Fig. 1a for actual units). Data are shown as mean ± SEM over all electrodes.

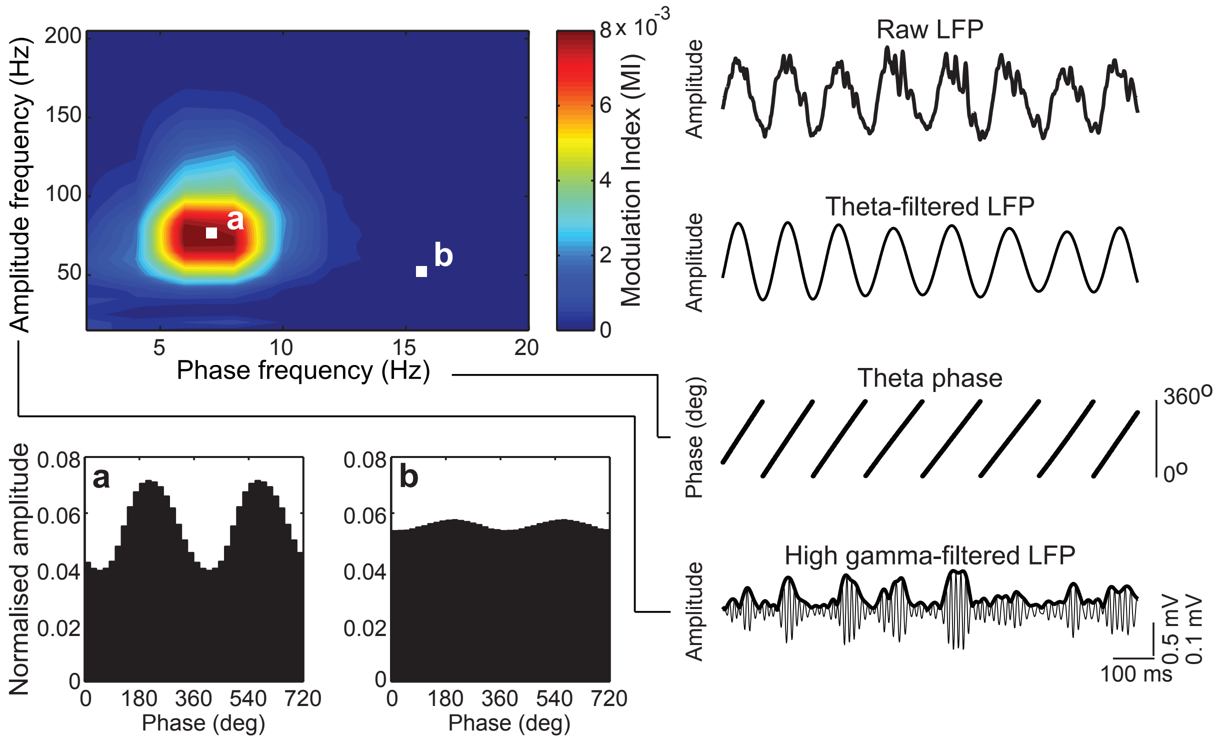

Supplementary Figure S3. Computing phase-amplitude comodulation maps. To compute each entry of the comodulation map (top left), the raw local field potential (LFP, top right) signal is band-pass filtered into two frequencies: a phase-modulating frequency (i.e. theta) and an amplitude-modulated frequency (i.e. high-gamma). Next, the phase (third row) and amplitude (fourth row, thick line) time series are calculated from each of the filtered signals and used to compute phase-amplitude distribution-like plots (bottom left). In this example, plot **a** shows the mean 80-Hz amplitude distribution over 20° phase bins of the 8-Hz oscillation, and **b** shows the mean 80-Hz distribution over 20° phase bins of the 16-Hz oscillation. The modulation index (MI) for each of these frequency pairs (8 Hz & 80 Hz and 16 Hz & 80 Hz) is a measure of divergence of the amplitude distribution from the uniform distribution (see ref. 22 for details). This procedure is repeated for several frequency pairs (2-20 Hz x 20-200 Hz), and the MI values for each pair are displayed in a pseudocolor comodulation map (top left). Notice that **a** has stronger coupling (and, therefore, higher MI values) than **b**. The example shown in this figure was obtained from a CA1 recording during active exploration (adapted, with permission, from ref. 23).

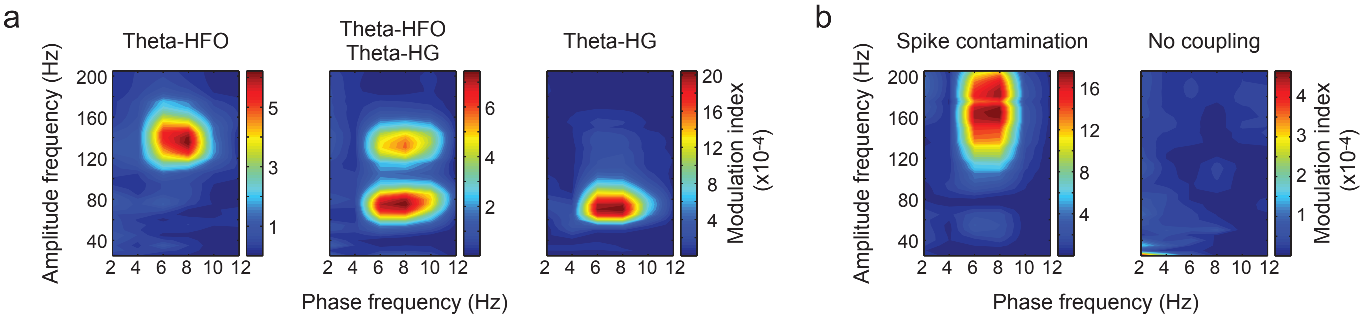

Supplementary Figure S4. Representative examples of electrodes either included or excluded from the cross-frequency coupling (CFC) strength analysis. (**a**) Examples of electrodes included in the CFC analysis. Theta-HFO coupling strength was only considered for electrodes that exhibited theta-HFO coupling in the comodulation map during baseline recordings, as is the case of the left and middle panels. Theta-HFO coupling was most apparent in electrodes located above the pyramidal layer (see refs. 23 and 39). Similarly, only electrodes that exhibited theta-HG coupling in the comodulation map (middle and right panels) were used in the analysis of theta-HG coupling strength. Theta-HG coupling was strongest in stratum lacunosum-moleculare (right panel, see also ref. 23), but could also be seen at lower levels in electrodes near the pyramidal layer along with theta-HFO coupling (middle panel). (**b**) Examples of electrodes excluded from the CFC analysis. Electrodes located at the CA1 pyramidal layer and in the dentate gyrus typically exhibited comodulation maps in which theta modulates a wide range of higher-frequency oscillations (left panel). This type of coupling has recently been shown to correspond to contamination of the LFP signal by multiunit activity (see refs. 30 and 39) and was discarded from the CFC strength analysis. Some electrodes were also not considered in the CFC strength analysis because they exhibited no coupling in the comodulation map (right panel).
